# Behavioral and trophic segregations help the Tahiti petrel to cope with the abundance of wedge-tailed shearwater when foraging in oligotrophic tropical waters

**DOI:** 10.1038/s41598-020-72206-0

**Published:** 2020-09-15

**Authors:** Andreas Ravache, Karen Bourgeois, Henri Weimerskirch, Angélique Pagenaud, Sophie de Grissac, Mark Miller, Sylvain Dromzée, Anne Lorrain, Valérie Allain, Paco Bustamante, Jonas Bylemans, Dianne Gleeson, Yves Letourneur, Éric Vidal

**Affiliations:** 1 UMR ENTROPIE (IRD, Université de La Réunion, CNRS, Université de La Nouvelle-Calédonie, Ifremer), Centre IRD Nouméa, BP A5, 98848 Nouméa, New Caledonia; 2 IMBE, Aix-Marseille Université, CNRS, IRD, Avignon Université, Centre IRD Nouméa, BP A5, 98848 Nouméa Cedex, New Caledonia; 3 Centres d’Etudes Biologiques de Chizé – CNRS, Villiers-en-Bois, France; 4 School of Biology, University of Leeds, Leeds, UK; 5 IRD, Univ Brest, CNRS, Ifremer, LEMAR, 29280 Plouzané, France; 6 The Pacific Community (SPC), BP D5, 98848 Nouméa, New Caledonia; 7 Littoral Environnement et Sociétés (LIENSs), UMR 7266, CNRS - La Rochelle Université, 2 Rue Olympe de Gouges, 17000 La Rochelle, France; 8 Institut Universitaire de France (IUF), 1 rue Descartes, 75005 Paris, France; 9 Institute for Applied Ecology, University of Canberra, Bruce, ACT Australia; 10 Department of Ecology and Evolution, Biophore, University of Lausanne, Lausanne, Switzerland; 11 UMR ENTROPIE (IRD, Université de La Réunion, CNRS, Université de La Nouvelle-Calédonie, Ifremer), Université de La Nouvelle-Calédonie, BP R4, 98851 Nouméa Cedex, New Caledonia

**Keywords:** Behavioural ecology, Stable isotope analysis, Conservation biology

## Abstract

Two species breeding in sympatry are more likely to coexist if their ecological niches are segregated either in time, space or in trophic habits. Here, we combined GPS-tracking, stable isotope analysis and DNA metabarcoding analysis to understand how the rare Tahiti petrel *Pseudobulweria rostrata* (TP) copes with the very abundant (i.e. 500,000 breeding pairs) wedge-tailed shearwater *Ardenna pacifica* (WTS) when breeding in sympatry in a tropical area. WTS foraged in restricted areas along their path, while TP predominantly foraged using extensive search behavior, suggesting a more opportunistic foraging strategy. Interspecific overlap of foraging areas was higher than intraspecific overlap. Breeding seasons largely overlap between species during the study, but TP seems to be asynchronous breeders. TP fed upon prey with higher δ^15^N values than WTS, and their diet was mainly composed of deep-sea organisms. TP could feed upon dead prey floating at the surface while WTS preyed mainly upon fish species that generally move in schools. Our study highlights several segregating mechanisms (temporal, behavioral and trophic) that could facilitate the coexistence of the two species despite the predominant number of WTS, and provides the very first information on the foraging and trophic ecology of the poorly-known TP.

## Introduction

The theory of ecological segregation postulates that coexisting species may partition their use of resources, in either time, space or trophic habits to avoid or limit competition, leading to niche divergence^1^. Among marine predators, seabirds provide good examples of ecologically similar coexisting species, sometimes breeding in sympatry and sharing foraging areas^2,3^. Inter- and intra-specific competition in seabirds are supposedly higher in tropical areas where marine productivity tends to be lower^4^ with a more patchy distribution of resources compared to temperate and polar areas^5^. It is particularly true during chick-rearing when breeding adults acts as central place foragers^6^. Competition for food resources is therefore expected to be particularly high for tropical seabirds during the breeding season.

The foraging ecology of Procellariiform seabirds (i.e. albatrosses, petrels and shearwaters) has been widely studied thanks to the development of tracking technologies such as miniaturised GPS loggers (e.g. ^7–9^). However, tropical regions remain overall poorly studied, despite being hotspots of seabird species richness^10^. Tracking data constitute a valuable tool to identify foraging areas, and to characterize foraging trips and at-sea behavior of birds. Information on seabird at-sea movements and behavior can be completed by complementary techniques such as stable isotope analyses (SIA), used to depict trophic ecology (e.g. ^11^). Carbon stable isotopes (^13^C/^12^C) provide information on the feeding habitat and resource use because they reflect the primary carbon sources within a food web^12^, while nitrogen stable isotopes (^15^N/^14^N), showing a stepwise enrichment at each trophic level, are used to estimate the trophic position^13^. Isotopic niche width (i.e. the isotopic composition of animal tissues in a multivariate space) is a powerful tool to investigate the ecological niche of the species studied^14^. At the population level, a wide isotope niche is typical of a generalist population, while a narrower isotope niche reveals a species specializing on a more specific trophic level and/or habitat. Additionally, SIA allow the assessment of temporal isotope variance among individuals by carefully selecting tissues with appropriate turn-overs, and examining the consistency of the isotope values among them^15^. Generalist individuals vary in their resource use, resulting in a wide isotopic niche of the population. However, specialist individuals have a consistent use of resources but variation among individuals would also result in a wide population isotopic niche. Finally, the prevalence of specialist individuals and low variation among individuals result in a specialist population, with a narrow isotope niche. Therefore, individual variation in resource use may influence the population dynamics and ecological interactions within and between species^16^. However, SIA alone provides a limited understanding of real trophic interactions, not allowing the proper identification of prey. Complementary molecular analyses such as DNA metabarcoding^17^ have been widely used in recent years to precisely investigate the diet of seabirds, including Procellariiform species, from feces or regurgitate samples. It allows for a semi-quantitative estimation of the food items^18–20^.

The Tahiti petrel (*Pseudobulweria rostrata*, hereafter TP) and the wedge-tailed shearwater (*Ardenna pacifica*, hereafter WTS), are two similar-sized Procellariid species breeding in the tropics, sometimes in sympatry. The WTS ranges throughout the Indian and Pacific Oceans, between latitudes 35°N and 35°S^21^. It is very abundant in the tropical Pacific^22^. WTS has been shown to use a bi-modal foraging strategy during chick-rearing, alternating a series of short trips close to the colony with longer trips over distant areas when surrounded by low-productivity environments^6,8,23,24^, supposedly in response to high competition for resources^25,26^. However, WTS can shift to a unimodal strategy when breeding in richer environments^27–29^. WTS is known to forage in multi species flocks, in association with sub-surface predators such as yellowfin (*Thunnus albacares*) and skipjack (*Katsuwonus pelamis*) tuna, which make epi- and meso-pelagic prey available at the surface^30–32^. WTS feed mostly upon cephalopods and fish usually by surface feeding, but can dive up to 66 m deep, with an average of 5–14m^22,33,34^. In the Southwestern Pacific, the breeding season of the species begins at the end of October with the return of breeding adults to colonies. Adults lay their eggs in December, and chicks fledge at the end of May.

On the other hand, the TP is a poorly known species since it is rarer, and has been poorly studied. Its population sizes are generally imprecise and speculative, estimated between 10.000 and 20.000 mature individuals worldwide^22,35^. This species is known to breed in French Polynesia, Fiji, American Samoa and New Caledonia (France)^35^. As TP movements at sea have never been tracked until this study, their foraging ecology is largely unknown. TP diet is suspected to be mostly composed of fish and cephalopods^32^. This species is an asynchronous breeder, with laying occurring throughout the year, but peaking at various periods of the year depending on the geographical area considered^36^. When breeding in sympatry, TP and WTS compete fiercely for nests^36^.

Here, we aim at understanding how the rare TP copes with the much more abundant WTS when breeding and foraging around New Caledonia. For this purpose, we combined GPS tracking data, SIA on blood samples, and DNA metabarcoding on regurgitate samples to depict their foraging and trophic ecology. We hypothesized that either temporal, spatial or trophic segregation would exist to reduce inter-species competition, especially during chick-rearing when the competition is likely to be maximum. If a temporal segregation occurs, we expect both species to have different breeding seasons, and/or to forage at different periods of the day. A spatial segregation would imply at least low overlap of core foraging areas while a trophic segregation would induce small isotopic niche overlap, and a difference in prey composition identified by DNA metabarcoding.

## Material and methods

### Study area

This study took place in New Caledonia, in the South-west Pacific, which is located in an oligotrophic area with low nutrient and low primary production. This area exhibits a large-scale north–south gradient, with salinity and temperature decreasing from north to south^38^.

New Caledonia is home of 500,000 WTS breeding pairs nest mostly on sandy islets while TP breeding pairs are rare and scarcely distributed. Indeed, it was tentatively estimated that 1000 TP breeding pairs were distributed across the ~ 300 km-long mountain ranges of the main island^39^ (Grande Terre) but this figure remains poorly supported by field data. In addition, 11 out of 70 islets surveyed in the southern lagoon houses a total of less than 100 TP breeding pairs^40^.

**Figure 1:**
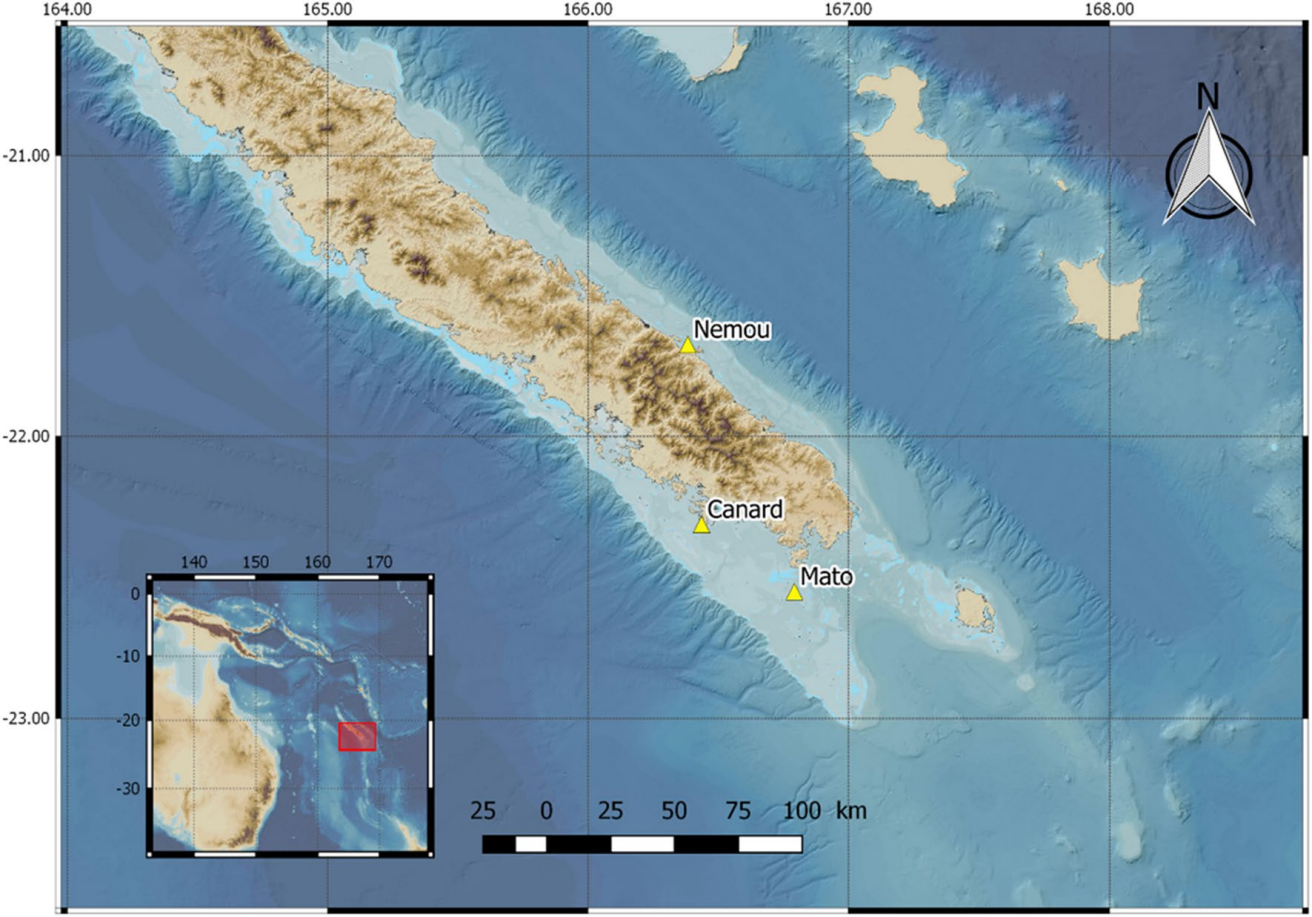
Location of the three study sites in New Caledonia: Nemou islet, a Tahiti petrel (TP) colony, Canard islet, a wedge-tailed shearwater (WTS) colony, and Mato, where WTS and TP breed in sympatry. The insert shows the position of New Caledonia in the South West Pacific. Loyalty Islands are visible at the top of the map. Bathymetry map was obtained from https://carto.gouv.nc/arcgis/services/fond_relief/MapServer/WMSServer. The map was created using QGIS version 2.18^41^ (URL: https://qgis.org/).

Study colonies are located on three lagoon islets situated off the southern part of Grande Terre (Fig. 1): Mato (22.55°S, 166.80°E), Canard (22.31°S, 166.31°E), and Nemou (20.38°S, 164.04°E). Mato and Canard are two close islets situated off the South-west coast of Grande Terre, while Nemou Islet is located off the South-east coast. Mato Islet hosts 2000 WTS and 20 TP breeding pairs. Canard Islet hosts 340 WTS breeding pairs. On Nemou Islet, 124 TP breeding pairs were recently censused (unpublished data).

### Field work

**Figure 2:**
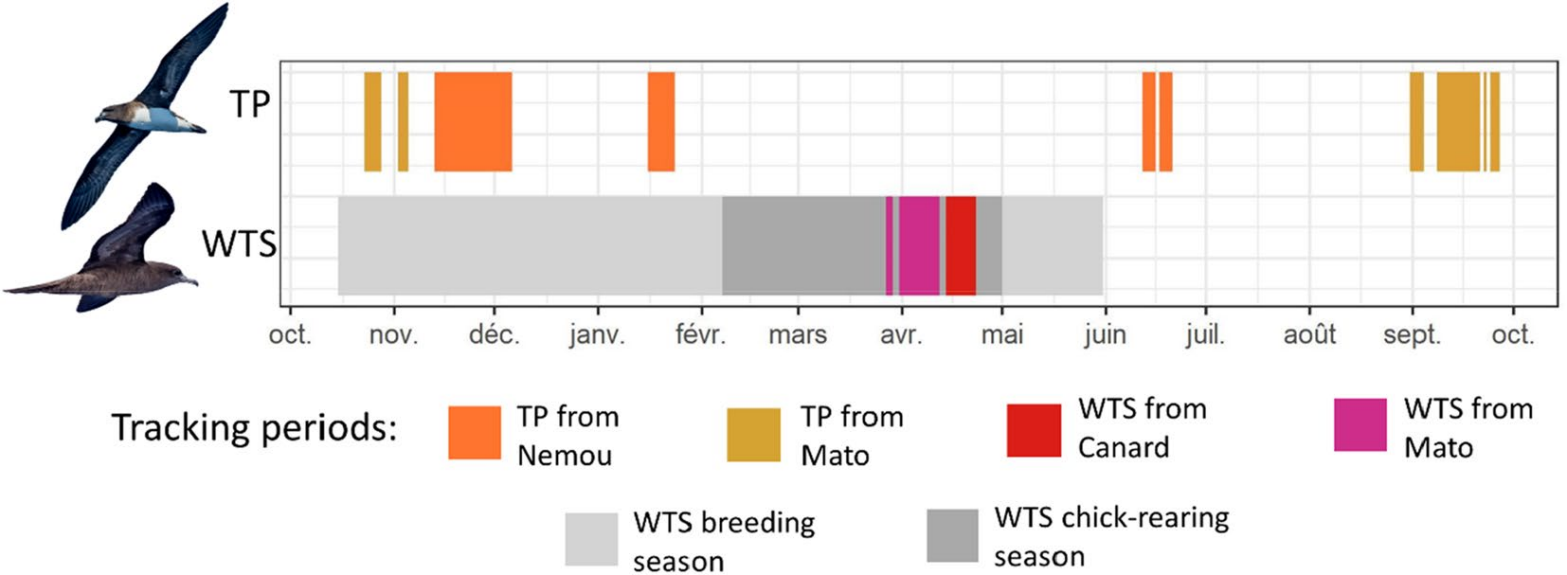
Periods of tracking for each species, and the extent of the breeding period of wedge-tailed shearwater (WTS) in New Caledonia, from the return of breeding adults to colonies (end of October) to chick fledging (May). Breeding and chick-rearing periods for Tahiti petrel (TP) are not shown as little prior information is available. Pictures are © Tubenoses Project, Hadoram Shirihai.

GPS-loggers were fitted on breeding adults during the chick-rearing period at the three study sites (Fig. 1). Phenology of individuals was determined by checking the presence of a chick in the burrow. On Mato Islet, 7 and 2 TP and 22 and 7 WTS were equipped in 2017 and 2018, respectively. On Canard Islet, 11 WTS were equipped in 2017. On Nemou Islet 3 and 9 TP were equipped in 2018 and 2019, respectively. Due to logistical and manpower constraints, the two species could not be tracked simultaneously, but 12 out of 27 TP were tracked during the WTS breeding season (mainly before the WTS chick-rearing phase; Fig. 2).

Breeding adults were fitted with either 4.5 g ECOTONE, 6 g LOTEK, or 12.5 g TECHNOSMART GPS-loggers, representing less than 3% of WTS and TP body weight (mean weight ± standard error: 413 ± 40 g and 430 ± 43 g in this study, respectively), i.e. below the limit commonly accepted to limit behavior modification^42^. The lightest GPS-loggers (ECOTONE and LOTEK) were attached to three tail feathers using TESA tape, while the heaviest GPS-loggers (TECHNOSMART) were back-mounted with 4 stripes of TESA tape to ensure that balance during flight would not be affected^43^. GPS were set to record location every 15 min. Birds were captured by hand at their burrow entrance before feeding their chick. Colonies were monitored every night for 15–20 days, to recapture birds for logger recovery. On recapture, blood was collected (maximum volume of 0.4 mL) from the tarsal vein using a 0.5 mL 29G syringe. Blood samples were centrifuged within 1 h from collection to separate plasma and blood cells that were then stored separately in 70% ethanol, and preserved in a cooler until return to the lab. During bird handling on Mato islet, spontaneous regurgitates were collected from 3 TP and 6 WTS and stored frozen at − 20 °C as soon as possible for DNA analyses. However, due to logistical constraints in the field, regurgitates were sometimes stored in a cooler for 1–3 days before being frozen. To estimate a possible impact of GPS-tracking on fledging success, we compared the proportion of TP chick-fledging in Nemou islet between 8 burrows were individuals were fitted with loggers and 45 control burrows. A chi-square test with Yate’s continuity correction indicated that GPS-equipment did not significantly impact the fledging success (Χ-squared = 0.023, *p* = 0.879).

### Phenology of Tahiti petrels

One hundred TP burrows were monitored every 2 months from July 2018 to March 2020 on Nemou Islet. Nest contents were checked using a burrowscope to determine the breeding status (i.e. egg, or stage of the chick). Camera traps placed in front of 15 burrows allowed to obtain the precise emergence and fledging dates of chicks. Combined with known incubation and chick-rearing duration (i.e. 55 days and 110–120 days respectively, according to Villard^36^), this allowed us to estimate egg-laying, hatching, and fledging dates with an accuracy ranging from 6 days to 2 weeks. Hatching dates were estimated by subtracting the average duration of chick-rearing to the fledging date and using the stage of development of the chicks. Stage of development was determined based on our knowledge of TP chick growth from prior monitoring (unpublished data) and coupled with information from prior studies^36^. Laying dates were estimated by subtracting the average incubation duration from the estimated hatching date. Egg-laying, hatching, and fledging dates were estimated similarly in burrows without camera traps, but with less accuracy, ranging from 2 to 3 weeks. For burrows containing a chick during our last visit (i.e. March 2020), the laying date was estimated by subtracting the incubation duration from the estimated hatching date. A total of 86 breeding events were observed during the study period. Egg-laying, hatching, and fledging dates were estimated and visually represented using 1d Kernel Density Estimate with the stat_density function implemented in the package ‘*ggplot2*’^44^.

### Foraging trip characteristics

Data for a total of 40 TP foraging trips were collected in 2017 (number of trips: n = 8), 2018 (n = 12), and 2019 (n = 20) from 21 GPS-tracked individuals, and 57 WTS foraging trips were obtained in 2017 (n = 45), and 2018 (n = 12) from 38 individuals. Multiple trips were sometimes recorded from the same individuals (Table 1).

**Table 1:** Table summarizing the number of repeated trips recorded per individual for each species.

Number of recorded tracks	Number of individuals	
	WTS	TP
1	24	11
2	11	5
3	1	3
4	2	0
5	0	2

The following metrics were calculated for each trip, using the R package “trip”^45^: (1) foraging trip duration from the departure to the return to the colony, (2) cumulative distance travelled between all locations assuming straight-line Euclidean distances between 2 successive locations, (3) maximum distance from the colony (hereafter “maximum range”), (4) average travel speed along the trip at sea (i.e. total distance travelled divided by the trip duration), and (5) maximum travel speed during the trip, computed between two successive locations, and assuming straight-line Euclidean distances. When tracks were incomplete (*i.e.* when the battery stopped before the individual started to return to the colony, n = 9), trip duration was estimated using the individual return date to the burrow surveyed by the field team. Total durations of six incomplete tracks were impossible to estimate, and these tracks were therefore removed from the trip duration analysis, resulting in a total of 53 and 24 trips being considered for WTS and TP respectively. Incomplete trips were also removed from maximal distance travelled analysis. To compare trip parameters between species, we constructed an ANOVA model including trip parameters as response variables (i.e. trip duration, distance travelled, maximum range, average speed and maximum speed) and species, colony type (i.e. uni-species or sympatric), interaction between species and colony type, and interaction between species and year as explanatory variables. Given the large-scale travelling capability of Procellariids seabirds^22^ and the proximity between colonies (i.e. less than 180 km apart by travelling over the sea), we did not expect the flight characteristics to be impacted by the fact that the two species are breeding in sympatry or in separate islets. However, colony type was kept in the analyses to make sure this assumptions are true. Pairwise Tukey HSD post-hoc tests were then conducted to test for differences between species. We examined the foraging trip length of breeding individuals using a frequency distribution of foraging trip duration for both species. Silverman’s test were performed to test if the distributions significantly differed from unimodal distribution. Moreover, as 3-day trip represents the same time than three 1-day trips, the frequency distribution plot underestimates the importance of long trips. Following Congdon et al.^24^, we calculated the proportion of time spent on trips of different length by each individual to remove this bias.

### Behavioral assessment and activity pattern

We determined TP and WTS at-sea behaviors using the Expectation Maximization binary Clustering (EMbC) algorithm^46^ for each species separately. EMbC is a robust multivariate clustering algorithm using speed and turning angle of the animal trajectory to identify four main behaviors. GPS tracks with intervals shorter than 30 min were analyzed using this method, and then each GPS location was assigned to a cluster (i.e. one of the four behaviors). Low speed and high turns were interpreted as intensive foraging, high speeds and high turns as extensive searching, low speeds and low turns as resting on the water and high speeds and low turns as travelling-commuting movements. This method has been used to assess ecologically meaningful behaviors from geolocation data for a range of seabird species, including Procellariiforms^47–51^.

In order to estimate daily activity patterns of each species, the total number of each behavioral type identified by the EMbC was summed per hour of the day, and divided by the total number of behaviors per hour, thus representing the relative proportion of each behavior according to the time of the day. Daily distribution of each behavior were compared using Watson-Wheelers test for homogeneity on two samples of circular data, implemented in the ‘circular’ package^52^. Proportion of each behavior was also calculated per individual in order to compare the time spent on each behavior between species. This comparison was computed using a multivariate analysis of variance (MANOVA), including the proportional use of each behavior per individual as response variables, and species, colony type (i.e. colonies where individuals breed in sympatry or where only one of the species breed) and their interaction as response variable. Finally, the proportion of each behavior were compared between daytime and nighttime for each species using a chi-squared test.

### Foraging areas and their overlap

To deal with data heterogeneity related to the use of various GPS devices with differences in the acquisition frequency of GPS locations (between 15 and 60 min, average 23.7 min for WTS, 27.0 min for TP), all tracks were interpolated at a regular interval of 15 min using the function redisltraj from the R package *adehabitatLT*^53^. Interpolated tracks were used to identify the main foraging areas of both species, by computing the Kernel Utilization Distribution of the GPS locations identified as “intensive foraging” or “extensive search” with a smoothing parameter h = 0.2° to avoid over-fragmentation, using the R package *adehabitatHR*^53^. Main foraging areas were defined as 90% Utilization Distributions (UDs), representing the 90 percent volume contours of the Kernel Utilization Distribution. Spatial overlap of colony foraging areas was determined by overlapping the UDs 90% of the different colonies for each species, and calculating the percent of area shared, ranging from 0–100% following this equation^54^:

$$\% \;{\text{Shared Area}} = \left[ {{\text{A}}_{0} } \right] \div \left[ {\left( {{\text{A}}_{{{\text{Colony}}1}} - {\text{A}}_{0} } \right) + \left( {{\text{A}}_{{{\text{Colony}}2}} - {\text{A}}_{0} } \right) + {\text{A}}_{0} } \right]$$

With A_0_ = the area of 90% UD intersection between colonies, and A_Colony_ = the area of the 90% UD of the colony, calculated with the package *rgeos*^55^.

### Depth of foraging bouts

The depth of the ocean where intensive foraging or extensive search bouts were performed was determined using the ‘ETOPO180’ variable downloaded from https://www.ngdc.noaa.gov/mgg/global/global.html. Values were compared between species using Wilcoxon–Mann–Whitney tests.

### Stable isotope analyses

Values of carbon (δ^13^C) and nitrogen (δ^15^N) were analyzed in plasma and red blood cells of GPS-tracked TP and WTS. Since lipids can affect plasma δ^13^C values, they were removed using 2:1 chloroform: methanol mixture^56^. Between 0.5 and 5 mg of dried plasma were repeatedly shaken (2–3 treatments) for 1 h in 4 ml of the solvent mixture. The sample was then centrifuged at 4000 g for 5 min and the supernatant containing the lipids was discarded. Lipid-free pellets were then dried at 60 °C overnight. Sub-samples of plasma and red blood cells were weighed (0.3 mg) with a microbalance, and packed into tin cups. Relative abundances of C and N isotopes were determined with a continuous flow mass spectrometer (THERMO SCIENTIFIC Delta V Advantage) coupled to an elemental analyzer (THERMO SCIENTIFIC Flash EA 1112). Replicate measurements of internal laboratory standards (acetanilide) indicated measurement errors < 0.10‰ for both δ^13^C and δ^15^N values. Stable isotope ratios are reported in δ (Delta) notation as parts per thousand (‰) deviation from the international standards δ^13^C_PDB_ and δ^15^N_air_ according to the equation $\updelta {\text{X }} = \left[ {{\text{Rsample }} / {\text{Rstandard}}} \right){-}1] \times 1000$ where X is ^13^C or ^15^N and R_sample_ and R_standard_ are the corresponding ratio ^13^C ⁄^12^C or ^15^N⁄^14^N of samples and international standards.

δ^15^N values are generally used as a proxy of the trophic level of consumers, and indirectly inform on the type of prey eaten^13^. We thus used them to test if TP and WTS forage at different trophic levels. δ^13^C values mainly reflect the carbon source food and habitat type of consumers^14,57^, and were used to look at a possible difference in foraging habitats (e.g. neritic vs. oceanic for instance) between the two seabird species. Stable isotope values were compared between species using a PERMANOVA, with δ^15^N and δ^13^C values as response variables, and species, colony type, year and as explanatory variables. As mentioned above, we did not expect an impact of colony type on stable isotope values, but this variable was kept in the analyses to ensure the veracity of our assumptions. The PERMANOVA was performed using the function adonis from the *vegan*^58^ package. SIA of carbon and nitrogen were used together to estimate and compare isotopic niche width between the two species using Stable Isotope Bayesian Ellipses In R (R package *SIBER*^59^). The standard ellipse area corrected for small sample sizes (SEA_C_), containing 40% of the bivariate δ^13^C and δ^15^N data, and the convex hull areas (TA) were computed for each species, giving an estimation of their isotopic niche width. For statistical comparison, we calculated the Bayesian standard ellipse areas (SEA_B_) from 10,000 iterations of Markov-chain Monte Carlo simulation^59^.

### Isotopic niche consistency

Plasma isotope values reflect diet integrated 3–4 days prior to sampling, whereas red blood cells isotopic values reflect longer term integrated diet (i.e. several weeks)^56^. Because of the different turn-over time between tissues, we used them to investigate the short-term consistency in the isotopic niche (i.e. consistency in trophic level and carbon sources) of both species by regressing stable isotope values in plasma on those in red blood cells^60^. Because δ^13^C has a trophic component, we used the studentized residuals of the relationships with δ^15^N in the same tissue to determine the degree of repeatability in δ13C, independently of trophic effects^61,62^. A significant result from the linear regression model would indicate the use of constant habitat (δ^13^C) or trophic level of prey consumed (δ^15^N) over time by individuals.

### DNA metabarcoding

Regurgitate samples were used for DNA metabarcoding dietary analyses. Only a brief overview of the DNA metabarcoding protocol is given below with the full details being described in the supplementary information. DNA was extracted from the regurgitate samples using a modified Qiagen DNeasy Blood & Tissue protocol. DNA extracts were screened for PCR inhibitors and the optimal working solution (i.e. undiluted DNA extracts or a 1:10 dilution) was used for the construction of high-throughput sequencing (HTS) libraries.

HTS libraries for each sample were constructed using fish (MiFish-U^63^), cephalopod (CephMLS^64^), and crustacean (CrustMLS^65^) specific primers with three PCR replicates performed for each sample by primer combination. Amplicon libraries were pooled and cleaned prior to paired-end sequencing at the Ramaciotti Centre for Genomics on the MiSeq platform using the v2 2 × 300 bp sequencing kit to obtain approximately 50,000–60,000 reads for each sample by primer combination. The *Trimmomatic* v.0.36^66^ and *OBITOOLS* software package^67^ were used for subsequent filtering of the reads following the general workflow described in De Barba et al.^68^. Taxonomic assignments were performed using both the approach available within the OBITOOLS pipeline and a BLAST search on the NCBI nucleotide database. A consensus taxonomic assignment was obtained considering only family and genus level assignments and used for further analyses. Negative extraction and PCR controls were used and carried through the workflow to assess potential cross-contamination and set minimal threshold values for species detections. Each prey taxa identified was categorized according to its habitat, depth range and migrating behavior, i.e., pelagic, benthic, benthopelagic, reef-associated, based on Fishbase^69^ and SeaLifeBase^70^ information.

The R packages *tidyverse*^71^ and *vegan*^58^ were used to summarize the data. A community matrix was constructed based on the presence-absence detection for the genus and family level assignments. A Bray–Curtis dissimilarity matrix was constructed, and used to produce a non-metric multidimensional scaling (NMDS) plot showing the dissimilarities in dietary composition between WTS and TP.

Data were compiled and analysed using R v3.4.0^72^.

### Ethical statement

All animal experimentation met the ABS/ASAB guidelines for ethical treatment of animals^37^. Permits to handle birds at studied sites were delivered and approved by New Caledonia’s Province Sud (permits nos. 609-2014/ARR/DENV, 2903-2015/ARR/DENV and 2695-2016/ARR/DENV).

## Results

### Phenology of Tahiti petrels

**Figure 3:**
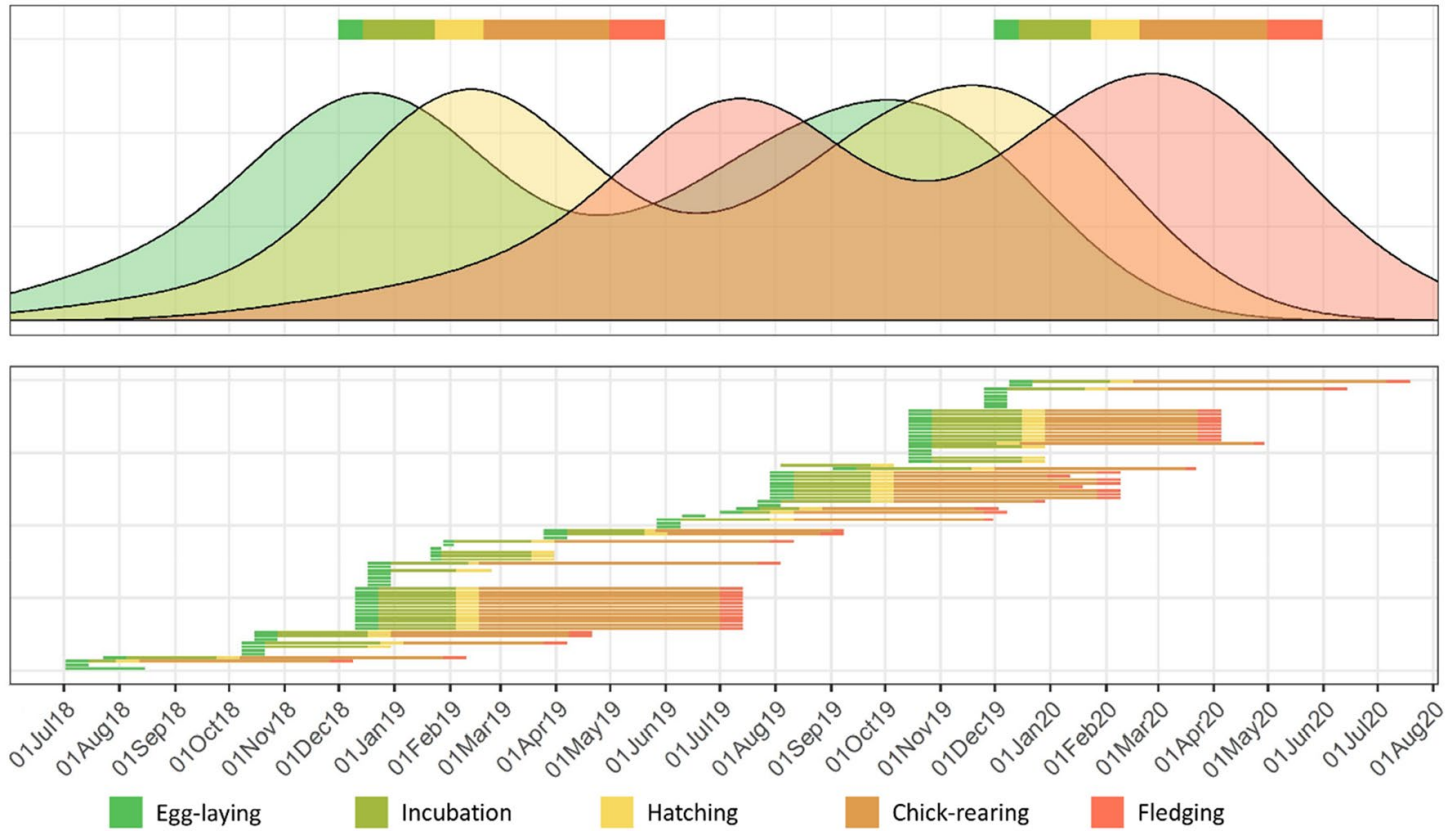
Top panel: Kernel density estimate of the various breeding periods observed for the Tahiti petrel (TP) on Nemou Islet from July 2018 to March 2020. The top colored rectangles represent the breeding season of wedge-tailed shearwater (WTS). Bottom panel: breeding periods of TP monitored on Nemou Islet. Each bar represents a burrow.

TP breeding period on Nemou Islet took place throughout the year, with egg-laying recorded during every visit to the islet. However, a first egg-laying peak occurred in December 2018, and a second one between September and October 2019 (Fig. 3). This led to peaks of hatching in February 2019 and November 2019. This implies that the two main chick-rearing periods spread from February to June 2019, and from December 2019 to April 2020. Therefore, TP main chick-rearing period largely overlapped with the WTS one in 2019 and, to a lesser extent, in 2020.

### Foraging trip characteristics

**Figure 4:**
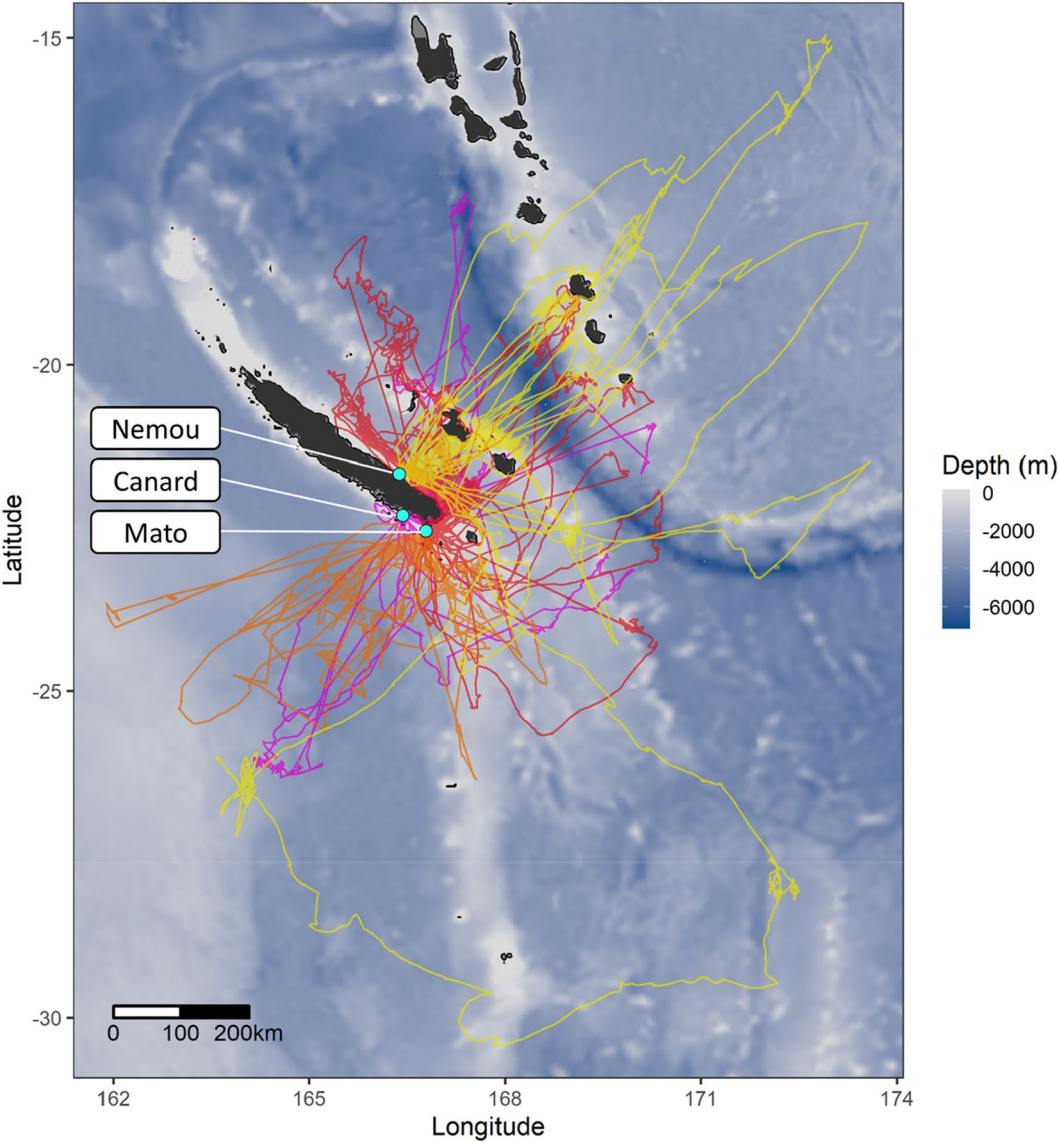
Foraging trips of GPS-tracked wedge-tailed shearwaters from Mato (red) and Canard (pink), and Tahiti petrel from Nemou (yellow) and Mato (orange). Blue dots represent the three study colonies. The three islands close to New Caledonia north coast are the Loyalty Islands, the islands further north are part of the Vanuatu archipelago. Bathymetry data were extracted from ‘ETOPO1 Global ReliefModel’ from ‘National Oceanic and Atmospheric Administration’. The map was created using the package ggplot2 version 3.3.2^44^ in R version 3.6^72^ (URL: https://www.r-project.org/index.html).

Trip duration (Anova: *p* = 0.600, Tukey post-hoc test: *p*.adj = 0.814) and maximum speed (Anova: *p* = 0.924, Tukey: *p*.ajd = 0.633) did not differ significantly between the two species (Table 2). In contrast, TP travelled significantly faster (mean speed, Anova: *p* < 0.001, Tukey: *p*.adj = 0.001), on longer trips (Anova: *p* = 0.021, Tukey: *p*.ajd = 0.005) and further from the colony (Anova: *p* = 0.031, Tukey: *p*.adj = 0.005) than WTS (Fig. 4).

**Table 2:** Numbers of GPS-tracked individuals, trips obtained and GPS-locations, and trip parameters of Tahiti petrel (TP) and wedge-tailed shearwater (WTS) from three colonies in New Caledonia. n represents the number of trips taken into account in the analysis. Adjusted *p* values are computed from Tukey HSD post-hoc tests, following an ANOVA. Significant *p*-values are marked bold.

	TP	WTS	Adjusted *p* value
Number of tracked individuals	21	38	
Number of foraging trips	40	57	
Number of GPS-locations before interpolation	6335	9915	
Number of GPS-locations after interpolation	7241	11,056	
Trip duration (days)	3.11 ± 0.34 (n = 37)	3.25 ± 0.41 (n = 53)	0.814
Distance travelled (km)	1151 ± 166 (n = 32)	654 ± 85 (n = 40)	**0.005**
Maximum range (km)	350 ± 40 (n = 38)	261 ± 21 (n = 50)	**0.005**
Average speed (km h^−1^)	15.9 ± 1.0 (n = 40)	12.6 ± 0.82 (n = 57)	**0.001**
Maximum speed (km h^−1^)	40.5 ± 1.1 (n = 40)	39.9 ± 1.0 (n = 57)	0.633

**Figure 5:**
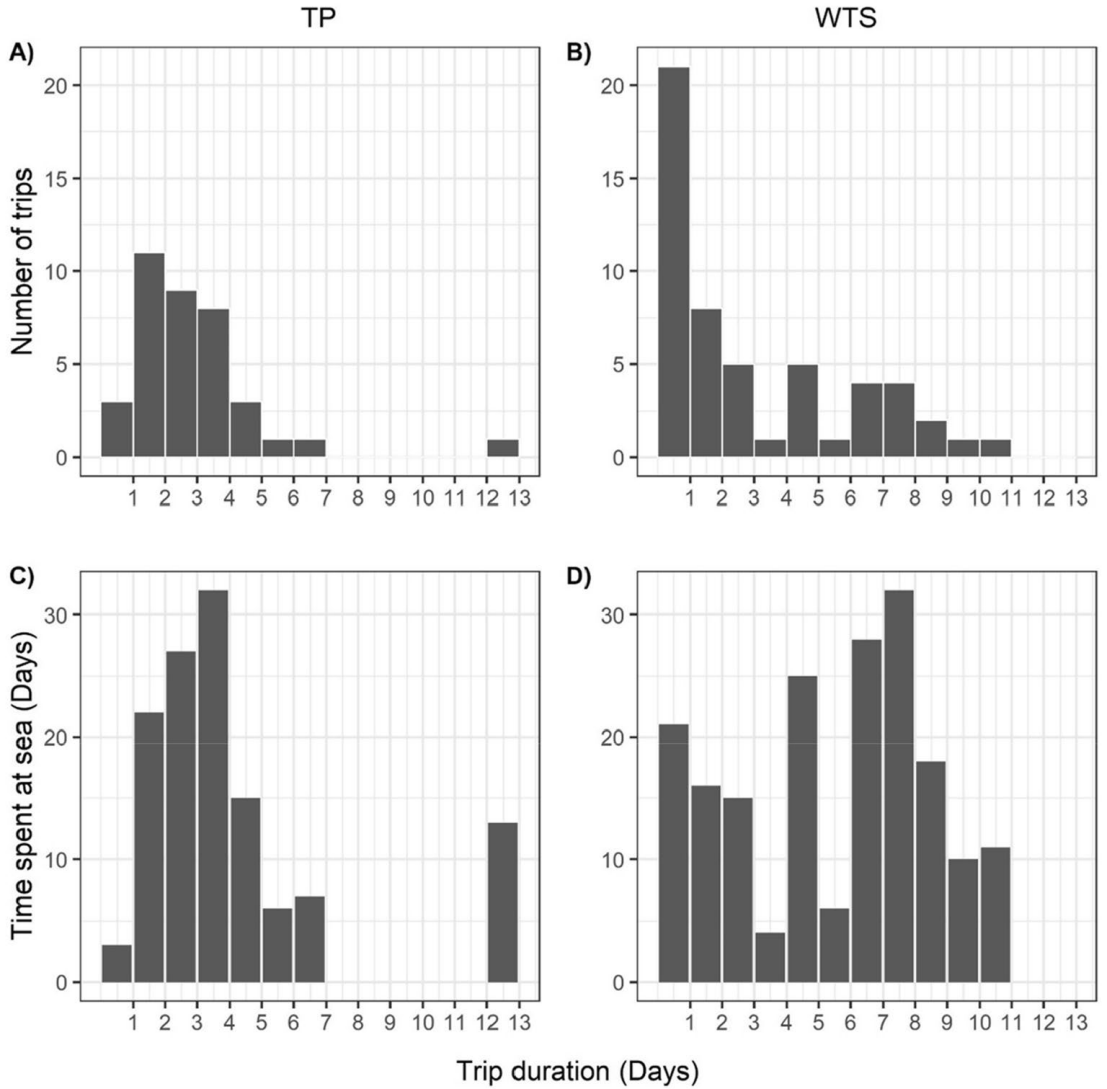
Distribution of foraging trip duration for Tahiti petrels (**A**) and wedge-tailed shearwaters (**B**). Time spent at sea during the foraging trips of TP (**C**) and WTS (**D**).

The frequency distribution of TP foraging trip duration suggests a unimodal foraging strategy with the majority of trips lasting 2 or 3 days (Fig. 5A), and most of the time spent at-sea being during 3-day trips (Fig. 5C). The frequency distribution of WTS trip duration indicates foraging trip duration ranging from 1 to 11 days with ~ 40% of 1-day trips (Fig. 5B). Silverman’s test indicated that TP and WTS foraging trip duration did not significantly differed from a unimodal distribution. However, when taking into account the time spent in each trip, WTS foraging trip duration visually appears bi-modal with most of the time spent at-sea during 1–3 and 7–8 days foraging trips (Fig. 5D).

### Behavioral assessment and activity pattern

**Figure 6:**
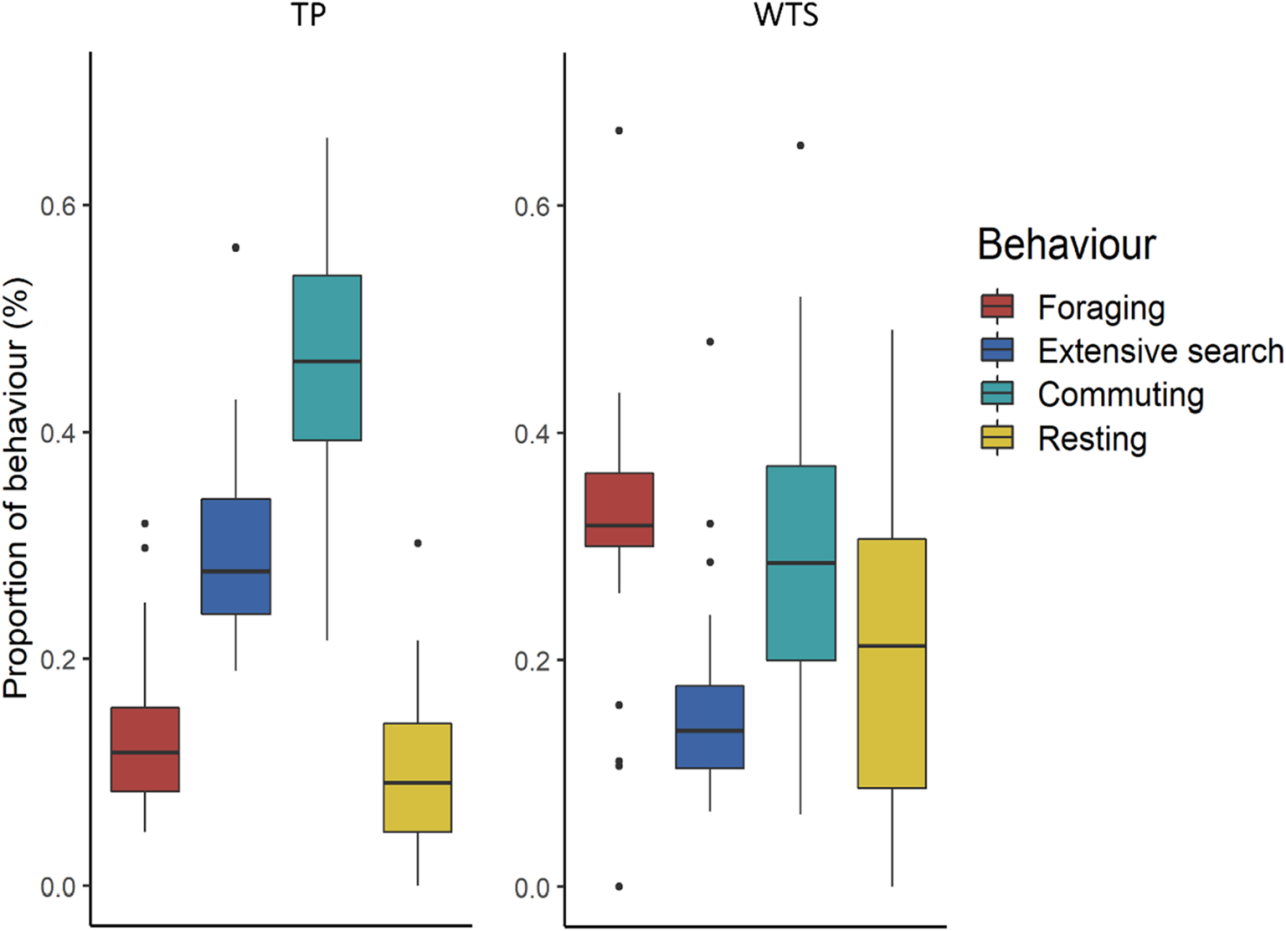
Proportion of the four behaviors assigned by EMbC along the tracks of Tahiti petrel (TP) and wedge-tailed shearwaters (WTS). Dots represent outliers.

The proportion of time allocated to each behavior as identified by EMbC, differed strongly between species according to the multivariate analysis of variance (*p* < 0.001), but they were no significant effects of the colony type (*p* = 0.64) or the interaction between species and colony type (*p* = 0.15; Fig. 6). TP spent significantly more time commuting (F = 20.9, *p* < 0.001) and searching extensively (F = 34.7, *p* < 0.001) but less time foraging intensively (F = 71.3, *p* < 0.001) and resting (F = 7.15, *p* = 0.009) compared with WTS.

**Figure 7:**
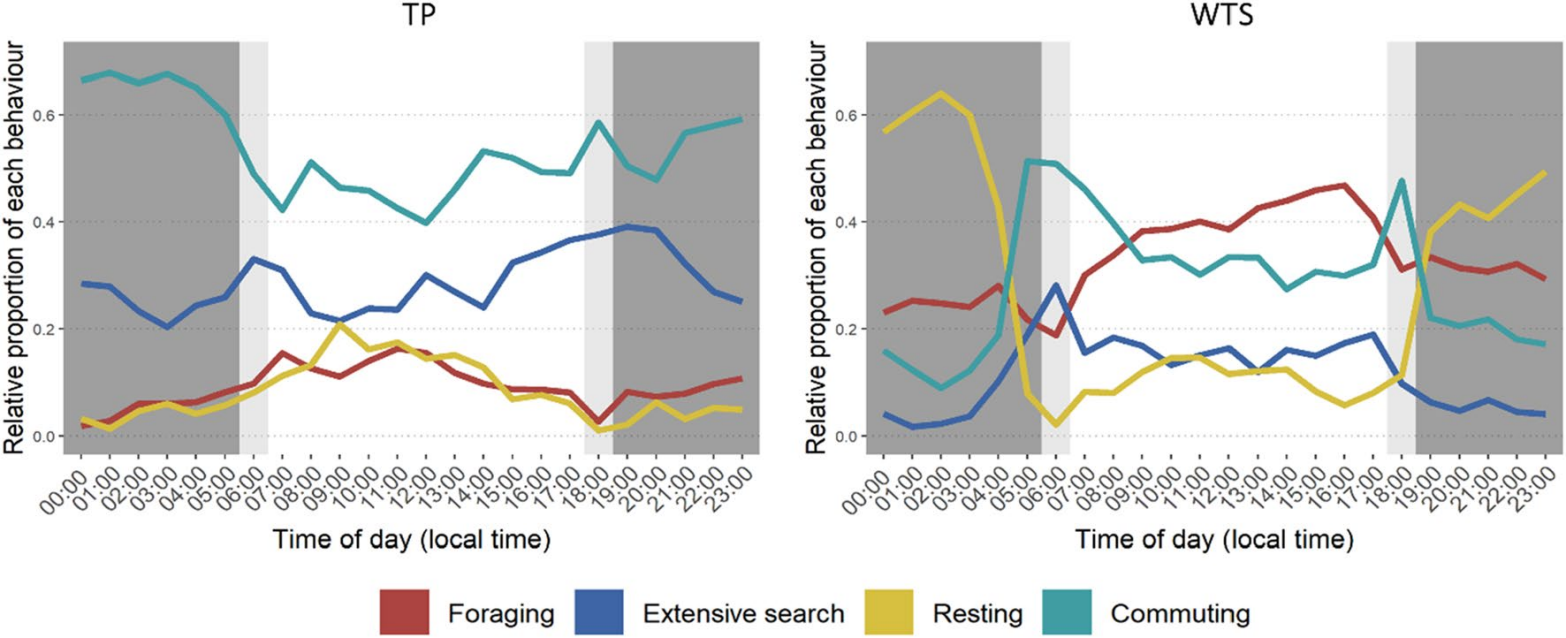
Percentage of time allocated to each behavior by breeding Tahiti petrels (left) and wedge-tailed shearwater (right), according to the time of day. The dark grey parts represent the night, the light grey parts the dawn and dusk, and the white part represents daytime.

The daily activity pattern also differed between species (Fig. 7). Watson–Wheeler tests indicated indicated that daily patterns of behavioural mode use were significantly different between species (foraging: W = 52.231, *p* < 0.001; extensive search: W = 104.14, *p* < 0.001; commuting: W = 109.96, *p* < 0.001; resting: W = 551.84, *p* < 0.001).

WTS behavior was clearly related to day/night cycle. The species performed intensive foraging mainly during the day (X-squared = 86.650, *p* < 0.001), rested mostly at night (X-squared = 2014.900, *p* < 0.001), and commuted at dawn and dusk. In contrast, TP showed a more consistent activity pattern between day and night. TP extensive search pattern was undifferentiated between day and night (X-squared = 0.416, *p* = 0.51), and intensive foraging was slightly more important during the day (X-squared = 24.487, *p* < 0.001).

### Foraging areas and overlap

All the TP trips from Mato Islet headed towards the south, while TP from Nemou Islet headed towards the north east, mostly targeting the coasts of Loyalty and Vanuatu islands (Fig. 4). WTS from Mato Islet mostly headed towards the east coast of New Caledonia, and WTS from Canard islet either towards the south or the north.

**Figure 8:**
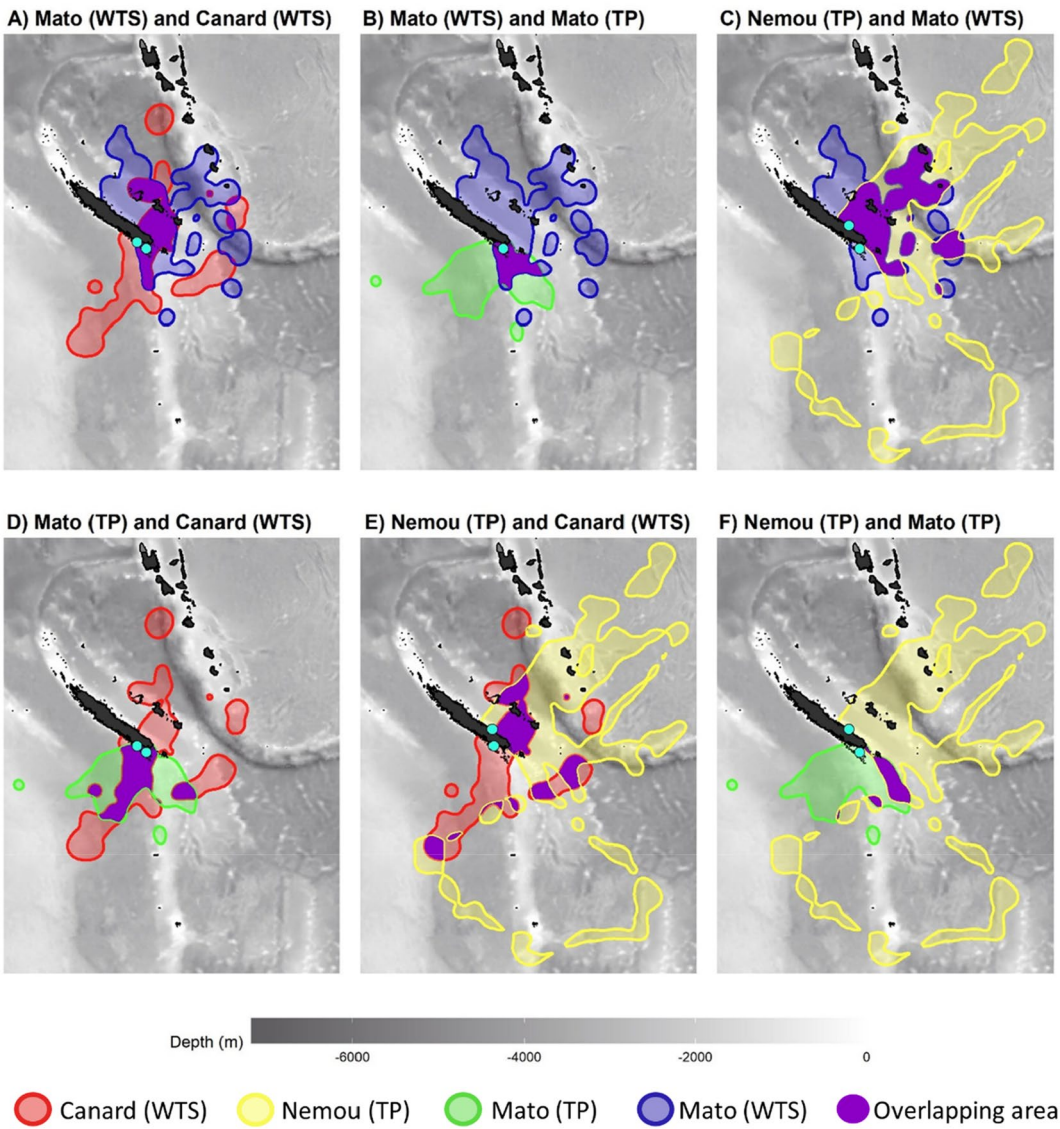
90% Utilization distributions (UDs) of foraging GPS locations of Tahiti petrels (TP) and wedge-tailed shearwater (WTS). The overlap between UDs 90% is represented in purple. Bathymetry data were extracted from ‘ETOPO1 Global ReliefModel’ from ‘National Oceanic and Atmospheric Administration’. The maps were created using the package ggplot2 version 3.3.2^44^ in R version 3.6^72^ (URL: https://www.r-project.org/index.html).

The inter-specific overlap of the foraging areas represented by the 90% Kernel Utilization Distribution was higher (mean: 15.1%) than the intra-specific (i.e. for individuals of the same species but from different colonies) overlap (mean: 9.7%, Fig. 8). However, where species are breeding in sympatry (Mato Islet), the overlap of foraging areas was low (7.8%) between species. Intra-specific overlap was higher in the WTS (16.2%) than in the TP (3.2%) (Table 3).

**Table 3:** Percentage of 90% UD overlap (see “Material and methods” section) among foraging areas of the three study colonies by species (*WTS* wedge-tailed shearwater, *TP* Tahiti petrel).

90% UDs overlap	TP Nemou	TP Mato	WTS Mato
TP Mato	3.2	–	
WTS Mato	21.0	7.8	–
WTS Canard	11.7	19.6	16.2

### Depth of foraging bouts

**Figure 9:**
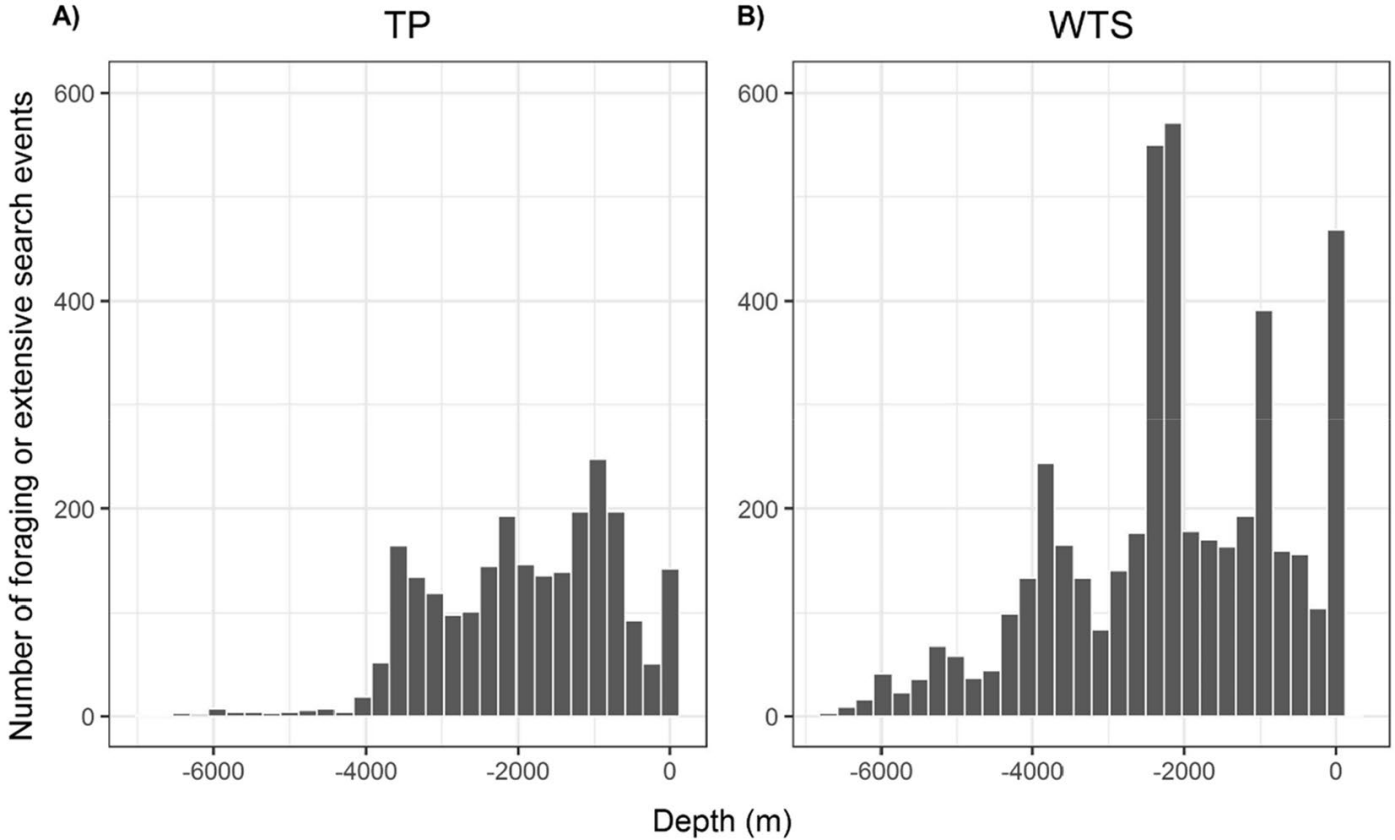
Frequency distribution of the depth of intensive foraging and extensive search bouts of Tahiti petrels (**A**) and wedge-tailed shearwaters (**B**).

Foraging locations of WTS were situated over significantly deeper areas than TP (mean depth: 2177 ± 22 m and 1900 ± 24 m, respectively, Wilcoxon test: W = 499,910, *p* < 0.001, Fig. 9). This difference remains significant (Wilcoxon test: W = 1,582,300, *p* < 0.001) when considering only birds breeding in sympatry (i.e. from Mato islet).

### Stable isotope analyses

The PERMANOVA indicated a significant effect of species (R^2^ = 0.86, *p* = 0.001), Year (R^2^ = 0.06, *p* = 0.001), and interaction between year and species (R^2^ = 0.02, *p* = 0.001) on δ^13^C and δ^15^N values. Colony type was not significant in this analysis (R^2^ = 0.003, *p* = 0.06), and neither was the interaction between species and the colony type.

**Figure 10:**
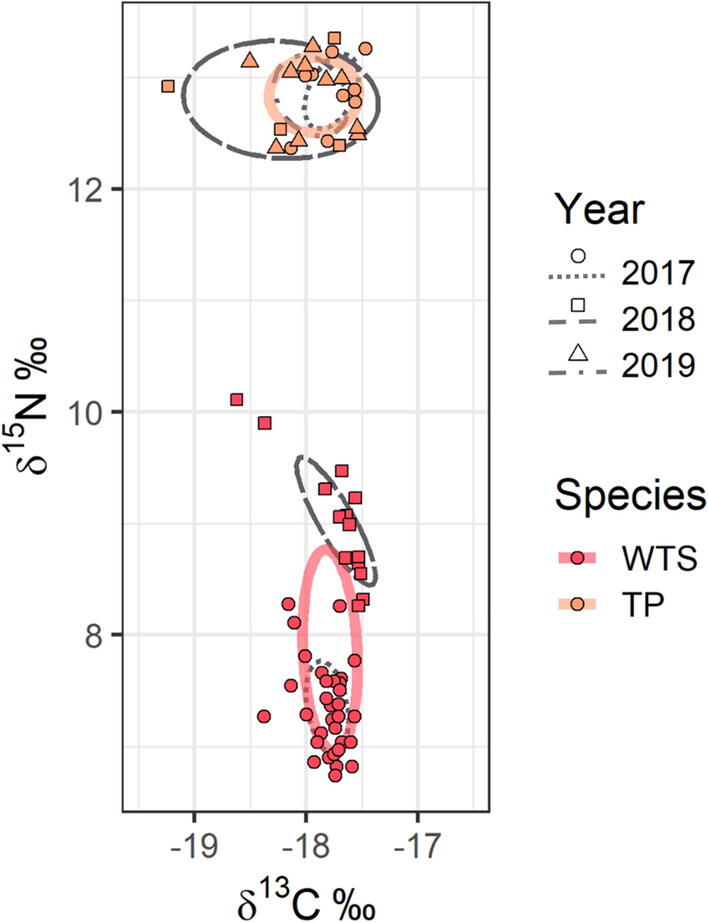
Isotopic bivariate niche space of Tahiti petrels (TP, orange triangles) and wedge-tailed shearwaters (WTS, red dots). SEA_C_ are represented by the solid bold lines.

Mean δ^13^C values in red blood cells were -17.93 ‰ (± 0.07, n = 23) for TP, and − 17.79 ‰ (± 0.04, n = 46) for WTS (Fig. 10), and did not differ significantly (W = 641.0, *p* = 0.156). Mean δ^13^C values for TP were − 17.772 ‰ ± 0.076 in 2017, − 18.227 ‰ ± 0.357 in 2018, and − 17.953 ‰ ± 0.099 in 2019. Mean δ^13^C values for WTS were − 17.812 ‰ ± 0.033 in 2017 and − 17.732 ‰ ± 0.091 in 2018.

In contrast, δ^15^N values were significantly different between species (W = 0.0, *p* < 0.001), with mean δ^15^N values in TP 5 ‰ higher than those in WTS (12.84‰ ± 0.07, n = 23 and 7.86‰ ± 0.13, n = 46; respectively). Mean δ^15^N values for TP were 12.872‰ ± 0.104 in 2017, 12.800‰ ± 0.215 in 2018, and 12.837‰ ± 0.107 in 2019. Mean δ^15^N values for WTS were 7.325‰ ± 0.072 in 2017 and 9.0.21‰ ± 0.147 in 2018.

Mean δ^13^C values in plasma were − 17.48 ‰ (± 0.06, n = 16) for TP and − 16.93 ‰ (± 0.03, n = 43) for WTS. Mean δ^15^N values in plasma were 13.53 ‰ (± 0.18, n = 16) for TP and 8.77‰ (± 0.11, n = 43) for WTS.

During chick-rearing, both species exhibited narrow, non-overlapping isotopic niches (Fig. 10, Table 4). However, WTS had a wider breadth of δ^15^N values, which resulted in a wider isotopic niche for this species (SEA_B_: *p* = 0.03).

**Table 4:** Total areas (TA), corrected standard ellipse areas (SEA_C_), and Bayesian standard ellipse areas (SEA_B_), all expressed in ‰^2^, of Tahiti petrels (TP) and wedge-tailed shearwaters (WTS) red blood cells.

	TP (n = 23)	WTS (n = 46)
TA (‰^2^)	1.088	2.608
SEA_C_ (‰^2^)	0.407	0.675
SEA_B_ (‰^2^)	0.392	0.624

### Isotopic niche consistency

**Figure 11:**
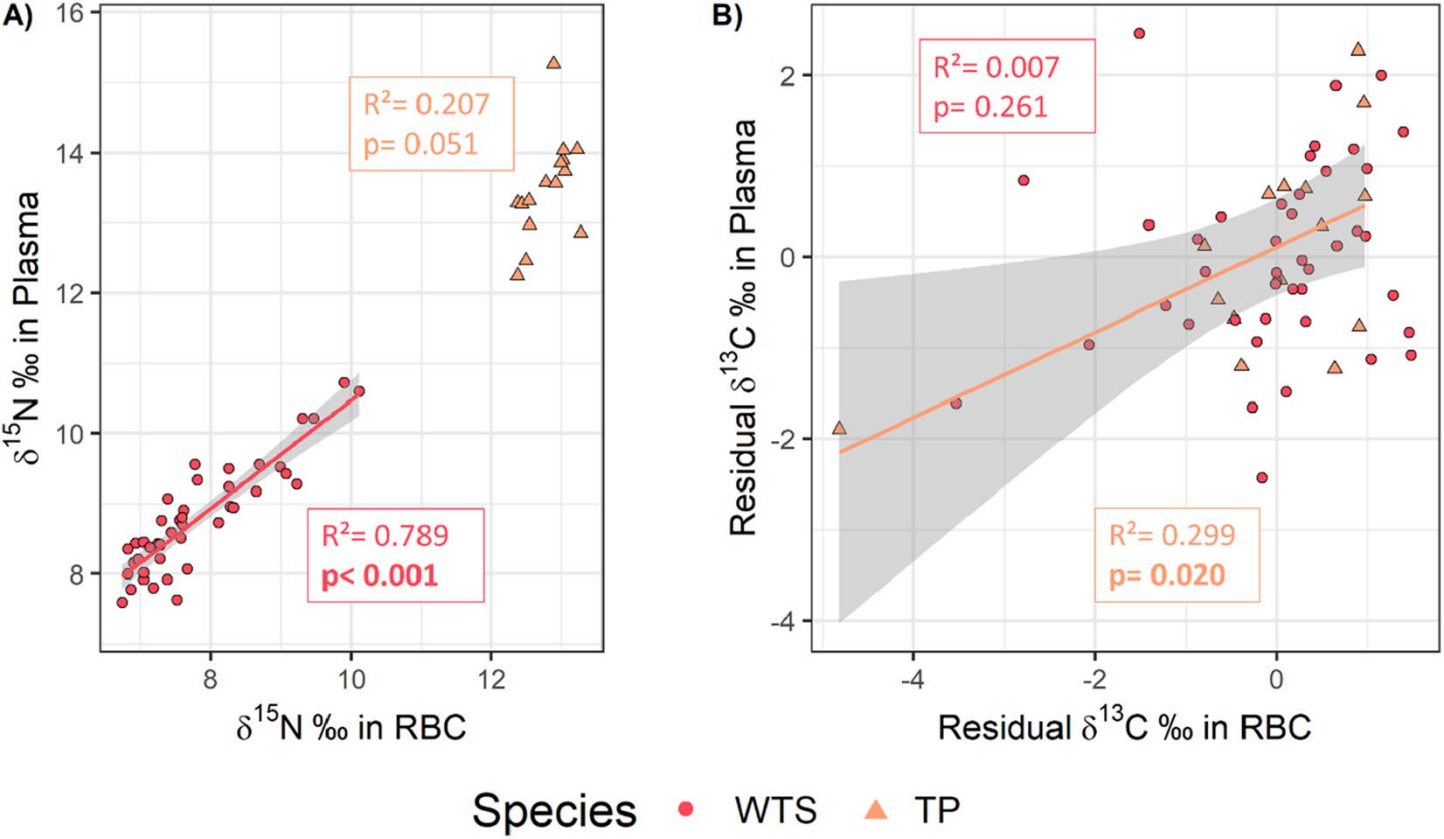
Relationship between δ^15^N (**A**) or residuals δ^13^C (**B**) values in red blood cells and plasma for Tahiti petrels (TP, orange) and wedge-tailed shearwaters (WTS, red). Lines indicate linear regressions and grey shadows their 95% confidence interval. R^2^ and *p* values of the models are represented in the boxes of corresponding colors. Significant *p* values are indicated in bold.

No significant relationship was found in δ^15^N values between red blood cells and plasma of TP (R^2^ = 0.207, *p* = 0.051, Fig. 11A), but a positive significant relationship was found in δ^13^C values (R^2^ = 0.299, *p* = 0.020, Fig. 11B), indicating short-term consistency of carbon source in TP. In contrast, no significant relationship was observed in WTS δ^13^C values (R^2^ = 0.007, *p* = 0.261), while δ^15^N values in WTS red blood cells and plasma were significantly positively related (R^2^ = 0.794, *p* < 0.001), indicating a short-term consistency in the trophic level of WTS prey.

### DNA metabarcoding

**Figure 12:**
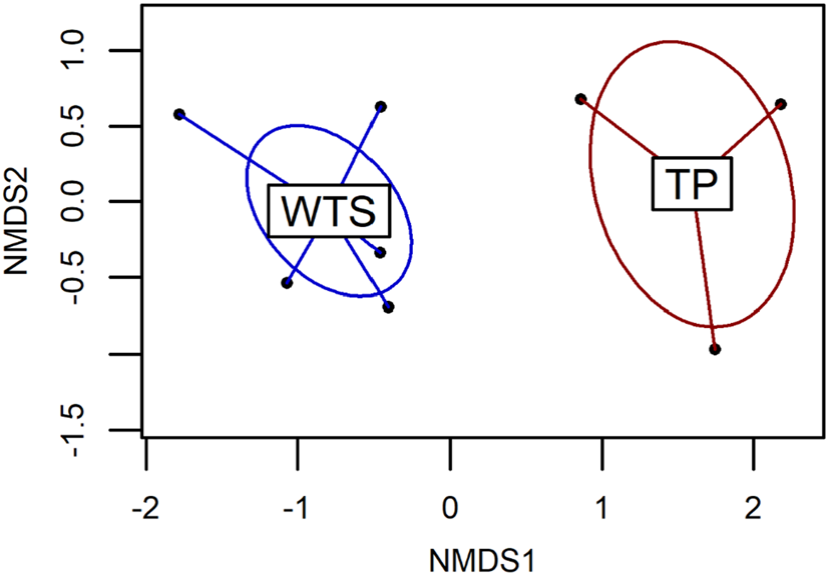
Non-Metric Multidimensional Scaling (NMDS) plot based on the presence/absence data derived from the DNA metabarcoding analyses of wedge-tailed shearwaters (WTS) and Tahiti petrels (TP) regurgitate samples. Dots represent the samples, ellipses show the clustering of the different samples according to species, and lines represent the distance of samples to the centre of the ellipse.

When considering the reads assigned to a family or genus level taxonomy, we obtained a total of 249,918 and 478,790 fish sequences, 146,962 and 55,068 cephalopod sequences and 94,046 and 284,686 crustacean sequences for TP and WTS respectively (see Supplementary Information S1 for summary data of the DNA metabarcoding analyses). A total of 18 taxa were identified in the 3 TP regurgitates with 6 fish families, 4 cephalopod families and 1 crustaceans family. Most of the prey were deep pelagic organisms migrating at the surface at night (Gempylidae, Myctophidae, Chiroteuthidae, Enoploteuthidae, Histopteuthidae, Onychoteuthidae, *Stylocheiron* sp.), but some of the prey were non-migrating deep pelagic organisms (Neoscopelidae, Sternoptychidae, *Euphausia* sp.), and three taxa of deep benthopelagic organisms were also identified (Macrouridae, Trichiuridae). Sequences obtained from the 6 WTS regurgitates allowed to identify 25 taxa including 5 fish families, 3 cephalopod families and 9 crustacean families. Fish prey were mainly pelagic species found close to the surface, noticeably anchovies *Encrasicholina* sp. found in the 6 samples, but also *Spratelloides* sp. (Clupeidae), *Decapterus* sp. (Carangidae), and the skipjack tuna *Katsuwonus* sp. (Scombridae). One fish (*Gempylus* sp.), the four cephalopod taxa observed (*Abralia* sp., *Enoploteuthis* sp., *Stenoteuthis* sp. and *Pterygioteuthis* sp.) and two crustaceans of the Euphausiidae family are deep pelagic organisms migrating at the surface at night. Most of the other crustaceans found in the regurgitates are benthic organisms (e.g. *Automate* sp., Axiidae) and some of them are reef-associated (e.g. *Alpheus* sp., *Dynomene* sp., *Atergatis* sp.). Of the limited number of regurgitate samples available, and over a total of 41 taxa identified overall, only 2 taxa (the cephalopod *Abralia* sp. and the krill *Euphausia* sp.) were found in the 2 seabird species regurgitates; the NMDS-plot showed a clear absence of overlap in prey species composition between TP and WTS (Fig. 12). The two-dimensional Bray Curtis dissimilarity index indicated a stress value of 9.72 × 10^–5^.

## Discussion

By combining information from GPS tracking, stable isotope analyses and metabarcoding, this study revealed how the rare and poorly known TP copes with the abundance of WTS foraging in oligotrophic tropical waters surrounding New Caledonia. These two similar-sized sympatric pelagic seabirds differ in their foraging strategy through behavioral differences and trophic segregation. In addition, this is the first comprehensive study to date on TP trophic ecology and foraging strategy, providing the first GPS tracking, isotopic niche and regurgitate metabarcoding data essential for the conservation of this species.

TP bred asynchronously on Nemou Islet, with breeding occurring throughout the year, and a chick-rearing period often largely overlapping the WTS chick-rearing season. Inter-specific breeding season overlap in seabirds has been previously shown to lead to competition for nests when breeding in sympatry, as previously evidenced in the New Caledonian Southern lagoon^36^. Such an overlap can also lead to competition for resources at-sea when sharing the same diet. Therefore, asynchronous breeding of TP implies only a partial seasonal segregation with WTS. At-sea activity patterns indicated that WTS mainly foraged by day, while TP foraged by day and by night, as previously suggested by Spear et al.^32^ from observations at sea. This partial temporal segregation of foraging activity may limit the competition for resources to some extent but could also be related to prey differences between the two species. Therefore, despite being not fully elucidated, partial seasonal and daily segregation could occur between species.

Overall, mean foraging trip duration did not differ between species, but the frequency of trip duration was differently distributed. This suggests a unimodal foraging strategy for the TP, and despite non-significant differences from uni-modality, a more bi-modal strategy for the WTS. The bi-modal foraging strategy of WTS breeding in New Caledonia has been previously demonstrated, and linked to poor local biological productivity, and potentially to strong intra-specific competition^8^. Indeed, studies conducted on breeding WTS proposed that adult foraging strategy is adjusted accordingly to the productivity of near colony areas^29^. Bimodal foraging strategies are assumed to be used by populations as a means of overcoming intra-specific limitations associated with insufficient resource availability near the colony^24,73,74^. In contrast, unimodal foraging strategies have been attributed to birds using only highly productive areas close to the colony^27,75^. Therefore, the unimodal foraging strategy exhibited by TP would suggest that they either access prey more efficiently, or have a different mode of foraging (travelling more, at higher speed).

Despite finding similar maximum flight speed between the species, TP travelled on average faster during their trips than WTS and travelled on longer distances suggesting contrasted behavior time allocations during foraging trips between species. Indeed, during foraging trips, TP spent more time commuting or extensively searching for food, and less time resting than WTS which mainly performed intensive foraging. Extensive search is characterized by high speed movements to forage in a large area in low detail in order to locate prey, while intensive foraging consists of low speed movements with high turning, covering a small area in detail once the individuals encountered areas where resources are plentiful (defined as Area Restricted Search^76^). This suggests that WTS forage mainly upon patchily distributed prey such as schools of fish, while TP exhibit a more opportunistic foraging behavior, travelling rapidly over longer distances to target more isolated prey.

Moreover, significant and substantial differences found in nitrogen isotope values between these two species suggest the consumption of different prey (e.g. different species and/or different size^77^). Despite the small sample size, DNA metabarcoding confirmed that WTS targeted schooling prey, such as Engraulidae or Clupeidae, which were the most frequent prey identified in regurgitates. The predation of such schooling species and their mainly diurnal foraging reinforce the hypothesis that WTS could have foraged in association with diurnal sub-surface predators^78^, making prey accessible to this shallow-diving species (mean maximum diving depth: 5–14 m^33,34^). Indeed, in Australia, WTS were documented foraging in association with yellowfin (*Thunnus albacares*) and skipjack (*Katsuwonus pelamis*) tuna^31,32^, which are mainly diurnal feeders^79^. WTS breeding in New Caledonia were shown to forage mostly over deep waters or more rarely at the edge of the barrier coral reef^8^. The presence of benthic crustaceans identified in their stomach could be secondary consumption, i.e. prey of species preyed by WTS, or could be the consumption of the pelagic larvae of those benthic adults. Moreover, as WTS can sometimes take advantage of the presence of moonlight to feed also at night^51^, it is possible that they caught some of the deep pelagic organisms (e.g. *Thysanopoda* sp., *Enoploteuthis* sp.) migrating to the surface at night.

On the other hand, the most frequent species in the stomach content of TP were deep pelagic fish and cephalopod species, migrating at the surface at night, and benthopelagic fish. Spear et al.^32^ found similar types of prey when visually analyzing and identifying stomach contents of TP caught at sea in the Eastern Tropical Pacific (i.e. presence of Sternoptychidae, Myctophidae, Macrouridae, Onychoteuthidae, Gempylidae, Trichiuridae and Histioteuthidae). The presence of benthopelagic prey in the stomach contents of TP (e.g.; Macrouridae, *Evoxymetopon* sp.) could partly explain the high δ^15^N values found in their red blood cells, as δ^15^N values of marine organisms, in some particular areas, may increase with the depth of their habitat^80^. Since TP do not dive, we can assume that deep prey performing diel vertical migration (e.g. Myctophidae) were captured at night, when coming closer to the surface. However, some deep-sea prey species of the TP do not undergo diel vertical migrations, and are thus unlikely to be present at the sea surface. An alternative explanation could thus be that TP scavenge on dead organisms floating at the surface, a behavior observed in many Procellariform species^81^. The high δ^15^N values and the high proportion of extensive search behavior in TP provide further support to this explanation. This is consistent with Spear & Ainley^82^ previous at-sea observations showing that TP obtained 100% of their prey by surface-seizing. This also supports the assumption of Spear et al.^32^ that 70% of squids eaten by TP were obtained by scavenging. Indeed, TP morphology (i.e. robust bill, extremely long tarsus, short tail, small wing area) is quite distinct from many other tropical petrel species, and Spear and Ainley^82^ supposed these characteristics to be morphological adaptations for ripping flesh from dead prey too large to be swallowed whole. They also considered their small wing area and short tail as additional adaptations allowing to efficiently search for non-active prey over large areas. Finally, higher δ^15^N values found in TP red blood cells might reflect the consumption of decomposing prey (since decomposing tissues undergo biochemical changes leading to higher δ^15^N values^83^), or alternatively, the consumption of larger prey, as δ^15^N values most often increase with organism size^77^, or prey situated at higher trophic levels^13^, all these hypotheses not being exclusive.

Our results also documented short-term consistency (within ca. 1 month) in the δ^15^N values of WTS, implying a greater variation in the δ^15^N values of prey consumed among individuals than within individuals. Along with the wider isotopic niche found in WTS compared to TP, and the broad range of δ^15^N values detected in their red blood cells, these results suggest that the New Caledonian WTS population is rather generalist, feeding on a variety of trophic levels, but composed of individuals differently specialized, which may reduce intra-specific competition^84^. In contrast, short-term consistency in δ^15^N values was not significant for TP. However, δ^15^N encompassed a shorter range, which could explain the absence of significant results, and suggest a specialist population in terms of trophic levels and/or origin of the prey consumed.

Short-term consistency in δ^13^C values was not significant in WTS, suggesting the use of variable habitats by the studied population. These results are consistent with their dual foraging strategy, alternating short trips over shallower waters and long trips over deep oceanic waters^8^, resulting in different δ^13^C values. In contrast, short-term consistency in δ^13^C values was significant in TP, suggesting the use of consistent foraging habitats within individuals, in line with the unimodal distribution of their trip duration. The use of constant foraging habitats could also partly explain the narrower isotopic niche width of the species. However, the model testing the δ^13^C values consistency poorly explained the deviance in these values, and these results have to be taken with precaution.

The high intra-specific segregation observed between WTS colonies found in this study seems to shape the at-sea distribution of the WTS population, most likely in response to the abundance of the species in the region^8^. Similarly, the two TP colonies are well segregated whereas inter-specific overlap is overall more important. This makes unclear the picture of a possible spatial segregation between TP and WTS. However, the depth of areas foraged is contrasted between the two species, with TP foraging over shallower waters. These results show that TP also forage in coastal areas, particularly individuals from Nemou Islet which travel along the coast of Loyalty Islands and Vanuatu, possibly in relation to their different diet. Tracking both species simultaneously and comparing other environmental variables such as sea surface temperature, productivity or distance to seamounts would be necessary to assess more finely the use of different habitats. This work is in progress and will be the subject of a forthcoming publication.

Finally, we showed that TP and WTS foraged in different habitats, but without a clear spatial segregation. Seasonal segregation occurs during a part of the year, as TP are asynchronous breeders, but most individuals from Nemou Islet were breeding during the WTS breeding season. Temporal segregation also takes place on a daily scale, TP foraging by day and by night, while WTS concentrating their activity during the day. Their diets differed widely, as shown by metabarcoding and stable isotope analyses. WTS mainly foraged on patchily distributed prey, possibly in association with sub-surface predators, while TP had a more opportunistic foraging behavior, and possibly often scavenged on dead prey floating to the surface. They could therefore be associated with fisheries discards. Thus, trophic segregation could facilitate TP access to food resources and the coexistence of the two species, despite the oligotrophic environment surrounding New Caledonia^38^, and the high abundance of WTS breeding in the area (i.e. > 500,000 breeding pairs^40^). By analyzing TP trophic ecology and foraging behavior for the first time, this study provides important information about this species relationship with prey, and crucial data for its conservation.

### Limitation of the study

Despite providing new insights in the TP ecology and the segregation occurring between species when breeding in sympatry, the present study shows some limitations. First of all, due to manpower constraints, the asynchronous breeding and the rarity of the Tahiti petrel, both species have not been tracked simultaneously. Thus, we cannot exclude that behavior and isotopic values were influenced by environmental conditions at the time of monitoring. Furthermore, we did not take the chick age into account in the models analyzing the trips characteristics. Seabirds can modify the length of their trips according to the breeding stage^85^. Thus, we cannot exclude that the very long trip (13 days) of one of the TP was linked to the advanced chick age. Finally, one of the limitation of the statistical analyses is that we could not include individuals as a random factor in the models. Given the low number of repeated tracks by individuals, including it as a random factor result in a singular fit of most of the models. However, precautions were taken to analyse the data in the most meaningful way possible, and clear patterns are emerging.

## Supplementary information

Supplementary Table.

Supplementary Information

## Data Availability

Data are available on reasonable request made to the corresponding author.
