# Supplementary material for: Behavioral and trophic segregations help the Tahiti petrel to cope with the abundance of wedge-tailed shearwater when foraging in oligotrophic tropical waters
Source: Sci Rep. 2020 Sep 15;10:15129. doi: 10.1038/s41598-020-72206-0 (PMC7492251; doi:10.1038/s41598-020-72206-0)
Supplement: Supplementary file 2 — Supplementary Information [file 41598_2020_72206_MOESM2_ESM.docx]

Behavioral and trophic segregations help the Tahiti petrel to cope with the abundance of wedge-tailed shearwater when foraging in oligotrophic tropical waters

**Andreas Ravache*^1,2^, Karen Bourgeois^2^, Henri Weimerskirch^3^, Angélique Pagenaud^1,2^, Sophie de Grissac^3^, Mark Miller^4^, Sylvain Dromzée^2^, Anne Lorrain^5^, Valérie Allain^6^, Paco Bustamante^7,8^, Jonas Bylemans^9,10^, Dianne Gleeson^9^, Yves Letourneur^11^, Éric Vidal^1^**

^1^UMR ENTROPIE (IRD, Université de La Réunion, CNRS, Université de la Nouvelle-Calédonie, Ifremer) and Labex « Corail », Université de la Nouvelle-Calédonie, BP R4, 98851 Nouméa Cedex, New Caledonia

^2^IMBE, Aix-Marseille Université, CNRS, IRD, Avignon Université, Centre IRD Nouméa - BP A5, 98848 Nouméa Cedex, New Caledonia

^3^Centres d’Etudes Biologiques de Chizé – CNRS, France

^4^School of Biology, University of Leeds, UK

^5^ IRD, Univ Brest, CNRS, Ifremer, LEMAR, F-29280, Plouzané, France

^6^Secretariat of the Pacific Community, SPC, BP D5, 98848 Nouméa Cedex, New Caledonia

^7^Littoral Environnement et Sociétés (LIENSs), UMR 7266 CNRS - La Rochelle Université, 2 Rue Olympe de Gouges, 17000, La Rochelle, France

^8^Institut Universitaire de France (IUF), 1 rue Descartes 75005 Paris, France

^9^Institute for Applied Ecology, University of Canberra, Bruce ACT, Australia

^10^Department of Ecology and Evolution, Biophore, University of Lausanne, Lausanne, Switzerland

^11^UMR ENTROPIE (IRD, Université de La Réunion, CNRS, Université de la Nouvelle-Calédonie, Ifremer) Université de la Nouvelle-Calédonie, BP R4, 98851 Nouméa cedex

Corresponding author:

Andreas Ravache: andreas.ravache@ird.fr

## DNA metabarcoding

Regurgitate samples (n = 9) were centrifuged at 7000 rpm for 10 minutes to pellet all the material. The supernatant was removed and the pellet was used for further DNA extraction using a modified Qiagen DNeasy Blood & Tissue protocol. Proteinase K (60 µL) and buffer ATL (560 µL) were added to the pellet and samples were incubated at 56°C for one hour. After digestion, 560 µL of buffer AL was added to the samples after which the manufacturer’s protocol was followed. For each batch extractions a negative control was included to test for potential cross-contamination.

DNA extracts from the regurgitate samples or negative extraction controls were first screened for PCR inhibition or for the presence of potential contaminant DNA, respectively. A SYBR®-green Real-Time PCR assay and fish specific metabarcoding primers (i.e. MiFish-U^1^) were used for this initial screening. For all regurgitate samples a 1:10 dilution of the DNA extracts was obtained and a single PCR replicate was run for each sample by dilution (neat and 1:10) combination. For all negative controls a total of three PCR replicates were performed for each undiluted DNA extract to assess the presence of potential contaminant DNA. The chemical constitution of the PCR reactions is consistent with Bylemans et al.^2^ and reactions were run on a BioRad CFX96 Real-Time PCR system. Thermal cycling conditions consisted of 5 min at 95°C; 50 3-step cycles of 30 sec at 95°C, 30 sec at 61.5°C and 1 min at 72°C; a final extension of 10 min at 72°C and a melting curve with a continuous increase of 0.05°C/sec from 60°C to 95°C. The optimal working solution for the regurgitate samples (i.e. neat or the 1:10 dilution) was determined based on the observed shifts in Ct-values, the shape of the amplification curve and the melt-curves. When positive amplification was observed in the negative extraction controls, those samples were included in the library construction for High-Throughput Sequencing (HTS).

Library construction for HTS used a one-step PCR amplification protocol with fusion tagged primers to simultaneously amplify the barcoding region and add technical sequences for HTS (i.e. sequencing primers and adaptors). For each sample sequencing libraries were constructed using fish (MiFish-U^1^), cephalopod (CephMLS^3^) and crustacean (CrustMLS^4^) specific primers. Unique combinations of both forward and reverse 7 bp Multiplex Identification (MID-) tag were used to uniquely label PCR amplicons from each sample. Triplicate PCR reactions were run for each sample by primer combination using the reaction conditions described previously^2^ and the above described thermal cycling profile adjusted for the primer specific annealing temperatures (i.e. 61.5°C for MiFish-U, 52°C for CephMLS and 49°C for CrustMLS). For each primer pair, amplicon libraries from 11-12 samples were subsequently pooled based on their average Ct-values. The volumes used to construct amplicon pools were determined based on the total number of positive PCR replicates per sample to ensure an equal representation of the amplicons from each sample. Amplicon library pools were cleaned using Agencourt AMPure XP Beads in a 1:1 volume ratio and amplicon concentrations for each pool were determined using a Qubit HS Assay. Next, a single amplicon library was constructed for each primer pair by combining equal amounts of amplicons from each pool. A final clean-up step was conducted for the three libraries and amplicon concentrations were determined as described previously. Finally, amplicons obtained from the three primer pairs were combined into a single library taking into consideration the numbers of samples for which libraries were constructed, the library concentrations and the expected length of each amplicon library. The final library was sent to the Ramaciotti Centre for Genomics for paired-end sequencing on the MiSeq platform using the v2 2x300bp sequencing kit to obtain approximately 50,000-60,000 reads for each sample by primer combination.

After HTS, the sequencing adaptors and sequencing primers were trimmed from the reads using Trimmomatic^5^ v.0.36. The OBITOOLS^6^ software package was used for subsequent filtering of the reads following the general workflow described in De Barba et al.^7^.The NGSFILTER script was used to assign sequences to the corresponding samples and the OBISPLIT script was used to create separate files for each sample. Unique sequences were clustered using the OBIUNIQ script before removing short sequences (i.e. remove sequences below 150 bp and 180 bp for the MiFish-U and CephMLS/CrustMLS primers respectively) and sequences with low occurrences (i.e. sequences with a count below 100). PCR and sequencing errors were removed using the OBICLEAN and OBIGREP scripts (i.e. remove all sequences identified as ‘internal’ by the OBICLEAN script). Subsequently, all sequences were combined into a single file and unique sequences were clustered while retaining the individual sample information. Finally, the ECOTAG script was used to assign taxonomic information to the sequences using a reference database build using the standard vertebrate and invertebrate sequences from the EMBL data repository. One of the downsides of the OBITOOLS pipeline is that the reference database build for taxonomic assignments requires the sequence information of the primer binding regions^6^ When metabarcoding primers overlap with commonly used barcoding primers the primer binding regions are often not available in the public data repositories. Consequently, sequence information for certain species may be missed during the building of the reference database which in turn negatively impacts the taxonomic assignments. To circumvent this problem, a BLAST search was performed for all sequence records using the NCBI nucleotide database. Sequences records were split according to the primers and a BLAST search was performed with the maximum target sequences set to 100 and with a minimum expected threshold of 1 x 10^-60^. BLAST assignments were further restricted to the taxonomic groups of interest for each primer pair (i.e. Actinopterygii, Cephalopoda and Crustacea for the MiFish-U, CephMLS and CrustMLS primers respectively). Based on the BLAST search a taxonomy was assigned to the sequences using a lowest common ancestor approach: firstly if a sequence had hits with an identity > 98% and all hits belong to the same species then a species level taxonomy was assigned, second if a sequence had hits with an identity > 95% and all hits belong to the same genus a genus level taxonomy was assigned, finally if a sequence had hits with an identity > 90% and all hits belong to the same family a family level taxonomy was assigned. Afterwards, both the OBITOOLS and BLAST assignments were imported into R version 3.4.1 and the data was merged into a consensus assignment. The taxonomic assignments of both approaches were compared and preference was given to the assignment with the lowest taxonomic rank. If both approaches produced taxonomic assignments with the same rank (e.g. to the species level) but the assignment were inconsistent, then the lowest common ancestor was assigned to the sequence read. Finally, sequence abundance data for the negative controls was evaluated and used to set minimum threshold values for the respective sequences obtained from the associated samples.

## References

1. Miya, M. *et al.* MiFish , a set of universal PCR primers for metabarcoding environmental DNA from fishes : detection of more than 230 subtropical marine species. *R. Soc. Open Sci.* **2**, 150088 (2015).

2. Bylemans, J., Gleeson, D. M., Hardy, C. M. & Furlan, E. Toward an ecoregion scale evaluation of eDNA metabarcoding primers: A case study for the freshwater fish biodiversity of the Murray–Darling Basin (Australia). *Ecol. Evol.* **8**, 8697–8712 (2018).

3. Jarman, S. N., Redd, K. S. & Gales, N. J. Group-specific primers for amplifying DNA sequences that identify Amphipoda, Cephalopoda, Echinodermata, Gastropoda, Isopoda, Ostracoda and Thoracica. *Mol. Ecol. Notes* **6**, 268–271 (2006).

4. Braley, M., Goldsworthy, S. D., Page, B., Steer, M. & Austin, J. J. Assessing morphological and DNA‐based diet analysis techniques in a generalist predator, the arrow squid Nototodarus gouldi. *Mol. Ecol. Resour.* **10**, 466–474 (2010).

5. Bolger, A. M., Lohse, M. & Usadel, B. Trimmomatic: a flexible trimmer for Illumina sequence data. *Bioinformatics* **30**, 2114–2120 (2014).

6. Boyer, F. *et al.* obitools: A unix‐inspired software package for DNA metabarcoding. *Mol. Ecol. Resour.* **16**, 176–182 (2016).

7. De Barba, M. *et al.* DNA metabarcoding multiplexing and validation of data accuracy for diet assessment: application to omnivorous diet. *Mol. Ecol. Resour.* **14**, 306–323 (2014).
